# Cardio-metabolic risks in preterm children: opportunities, cautions and the importance of longer-term study

**DOI:** 10.1038/s41390-026-04905-9

**Published:** 2026-03-17

**Authors:** Hanis Hidayu Kasim, Raul Chavez-Valdez, Divyen K. Shah

**Affiliations:** 1 Basic Medical Science 1, University Sains Islam Malaysia, Negeri Sembilan, Malaysia; 2 Division of Neonatology, Johns Hopkins University - School of Medicine, Baltimore, Maryland UK; 3 Neonatal Unit, Royal London Hospital, Barts Health NHS Trust, London, UK; 4 Centre for Neuroscience, Surgery and Trauma, Blizard Institute, Queen Mary University of London, London, UK

## Abstract

What does this article add to the existing literature:

- This commentary highlights the significance of conducting prospective research in preterm populations as they age to characterize growth trajectories and links with metabolic profiles, supporting the concept of the developmental origins of health and disease (DOHaD).
- Significant gaps in foundational knowledge on physiological responses and optimal metabolic demands in preterm children exist and constrain the depth of current interpretations.
- Methodological challenges of different anthropometric and metabolic assessment methods must be carefully weighed to ensure reliable conclusions for future clinical application.

Almost four decades ago, David Barker and colleagues, postulated the fetal and infant origins of adult diseases model after careful follow up and study of historical cohorts.^1^ This model alluded to the importance of programming by the “environment” in fetal and infant life, which was associated with cardiovascular diseases in later life. We now know that not just low birth weight but also being born preterm is associated with risks of cardiovascular disease and metabolic syndrome in later life.

With increased survival of preterm infants, the consequences of prematurity become more apparent and pose a substantial public health emergency. Although, children born preterm are prone to being overweight and obesity, as well as hypertension,^2^ the list of longer-term challenges and consequences of prematurity continue to expand.

Overall, the rates of overweight/obesity in all children and adolescents in the USA continue to rise.^3^ Data shows that young people of Hispanic ethnic background are at a higher risk of obesity than other ethnic groups, with the reasons being complex.^4^

In this issue of Pediatric Research, Rosenfeld et al.^5^ report on a follow up of 62 children of an original cohort of 120 children born at a mean gestation of 28 weeks to a mean age of 11 years and compared against a term matched cohort for multiple parameters. Rosenfeld et al.^5^ assessed multiple clinically relevant parameters focused on the early cardio-renal-metabolic risk in the preterm cohort (lipids, leptin, resistin, adiponectin, TNF-α, IL-6), which were followed at age 10–13, at which time they were compared against the term control group. The study design allowed assessment of intra- and inter-group changes that highlight physiological changes in the preterm population and may highlight potential markers that distinguish metabolic development between children born preterm vs. full-term.

Clinical studies comparing preterm cohorts with term-born controls assume that the term cohort represents a standard of healthy growth and development. However, given the lack of preterm‑specific reference thresholds for biological markers, the differences between the groups may reflect either true pathological signs or alternatively, a unique metabolic profile that continues to evolve beyond the first 1000 days of life. From a physiological perspective, measurements may lie outside term-based reference intervals yet still represent normal levels for this population, matching their unique metabolic and organ demands. This dilemma enhances the necessity to map the metabolic profile in preterm cohorts until a yet unknown age to determine the biological ranges specific to this population.

In this preterm birth (PTB) cohort although weight gain velocity was similar to the control cohort of children born at term, it was associated with higher rates of elevated systolic blood pressure (SBP) ≥ 90th centile and diagnosis of overweight/obesity compared to control children born at term. At age 10–13, the PTB cohort also demonstrated higher triglycerides and non-HDL cholesterol levels than the term cohort and longitudinally, the PTB cohort had serum leptin and leptin : adiponectin levels, which increased by ≥4-fold by 10–13 years. Of note, PTB female teenagers were more affected than males, with a suggested stronger association with diagnoses of overweight/obesity and elevated/hypertensive systolic blood pressure.

This research group has shown the consistent risk demonstrated in preterm population from year one of life through 13 years linking distinctive metabolic and inflammatory markers with normal growth gain pattern. The disproportionate cardio-metabolic response may support the notion that prematurity is associated with long-term alterations in metabolic programming, leading to maladaptive responses that diverge from their peer counterparts (control cohort), primarily observed in elevated/hypertensive SBP.

Recruitment of, and maintaining active follow-up of preterm children is undeniably challenging. Obtaining longitudinal samples in 59–62 preterm participants, therefore, represents a notable achievement and adds considerable value to the study conclusions. However, studies such as these have several limitations including relatively small numbers, possible selection bias, and in this case, the controls from a different cohort. In comparison cohort studies, the laboratory techniques for inflammatory and metabolic markers have been described in more detail. Although both datasets used standardized clinical assays, the analytic method/batch differences could contribute to inter-group difference, beyond true biological differences.^6,7^

The findings obtained by Rosenfeld et al.^5^ are important and may be concerning on multiple levels. Although there are overall worrying trends in increasing overweight/obesity in all our young people globally, the findings from this study demonstrate that children born preterm may be at a much higher risk of abnormal lipid metabolism and cardiovascular disease. The presence of elevated TG in preterm children with higher weight‑gain velocity and greater central adiposity is consistent with an evolving metabolic syndrome phenotype, which may warrant regular monitoring and early intervention or prevention.

However, further interpretation is complicated by the strong influence of feeding practices on lipid and metabolic parameters, particularly in children, where the mode of feeding is not controlled by the study design. Consequently, single time-point samples may not be reliable especially for lipids, as values can be highly-dependent on diet and may not fully capture the dynamic metabolic trajectory of preterm children. Integrating lipidomics into longitudinal studies, even with single sampling points at each visit could enhance depth of interpretation by capturing patterns across groups of lipid species rather than relying on a few conventional lipid measures alone.^8^

At the same time, studies in growing children must exercise caution in their choice of anthropometric indices. In regular anthropometric practices, the optimal metric depends on whether growth is being assessed cross‑sectionally or longitudinally.^9^ Weight gain velocity is a strong and clinically relevant parameter for capturing dynamic changes in growth rate over time.^10^ Standard deviation scores, and z‑scores for single parameters such as height and weight are often preferable to BMI z‑scores, especially in cohorts with a high prevalence of overweight and obesity, because BMI z‑scores can be less reliable for tracking change especially in percent body fat.^11^

With 86% of the PTB cohort of white Hispanic origin it is not possible to infer from this study whether these findings can be generalizable to all children born preterm or whether they have a propensity for children of white Hispanic origin. Ethnicity and populational-based study has a substantial impact on metabolic profiles, largely through extrinsic factors such as dietary patterns, lifestyle, and sociocultural context and by intrinsic factors such as gut microbiota composition.^12^ Furthermore, the psychology involved in caring for a “fragile” child, like those born preterm and even more so if they also have comorbidities (e.g., bronchopulmonary dysplasia), may result in limited active-life style and increased caloric intake. Therefore, conducting multicentre studies across diverse ethnic groups, controlling for all nutritional, medical and psychological, including social determinants of health, will be critical in enriching reference data and clarifying how these differences modify cardio‑metabolic risk in preterm and term-born populations.

Being born preterm has far-reaching consequences for ongoing risk of morbidities and disease that continue beyond the neonatal period. Further research is required to identify target populations and interventions in the neonatal period that may attenuate adverse outcomes. Similarly, work like Rosenfeld et al.^5^ may highlight the need for support and interventions during childhood targeted at at-risk groups in order to attenuate outcomes into adulthood.

The authors should be commended on their perseverance in following up this cohort. Studies such as these highlight the importance of longer-term vision and follow-up. Appropriate funding and resources are required to maintain databases, biobanks, and engagement with families. As well as asking the right questions, the onus is on us as medical researchers to ensure scientific rigor in interpretation of the results. We owe it to the families who consent to participate in this research to obtain as much value from the data as possible. The findings from such work can then not only inform the care we provide in the neonatal period, but also future research and policy to improve outcomes. From their earlier studies, Barker and colleagues demonstrated the value of historic data and long-term follow-up in understanding the origin of health and diseases through early life determinants. Applying this principle in the current world can bridge the gap in identifying early changes with potential for early intervention and targeted clinical monitoring, with the aim to anticipate and attenuate long-term health outcomes in high-risk groups such as preterm populations.
